# What’s New in Neuropathy?

**DOI:** 10.7759/cureus.44952

**Published:** 2023-09-09

**Authors:** Giustino Varrassi, Stefano Tamburin, Panagiotis Zis, Vittorio A Guardamagna, Antonella Paladini, Martina Rekatsina

**Affiliations:** 1 Department of Pain Medicine, Paolo Procacci Foundation, Rome, ITA; 2 Department of Neurology, University of Verona, Verona, ITA; 3 Department of Neurology, University of Cyprus, Nicosia, CYP; 4 Department of Anesthesia, European Institute of Oncology (IEO), Milano, ITA; 5 Department of Life, Health and Environmental Sciences (MESVA), University of L'Aquila, L'Aquila, ITA; 6 Department of Anesthesiology and Pain Management, University of Athens, Athens, GRC

**Keywords:** neuropathic pain registry, neuropathy, neuropathic pain, gluten neuropathy, diagnosis of neuropathy

## Abstract

Neuropathic pain presents diagnostic and treatment challenges. Despite recent advances in our understanding of the diagnosis and treatment of neuropathy, much remains to be elucidated. Familiar with neuropathy is the paradox that aberrant nerve signaling causes both sensory loss and pain. Voltage-gated sodium channels play an important role in neuronal electrogenesis and communication among neurons, and their dysregulation leads to hyperexcitability and pain. While numerous validated diagnostic assessment tools are available for neuropathy, patients often experience a diagnostic delay about the cause of their neuropathy. New research is defining more specific types of neuropathy beyond peripheral and central forms. The prevalence of pain varies by type of neuropathy, with chronic idiopathic axonal polyneuropathy associated with the highest proportion of patients experiencing pain. In the majority of types, it exceeds 50%. Gluten neuropathy, a form of peripheral neuropathy, is a new diagnostic consideration. It may require electrochemical conductance testing of hands and feet to test for sudomotor dysfunction. Among those with serologically confirmed gluten sensitivity or celiac disease, gluten neuropathy is a common neurological manifestation and may be addressed at least partially by a gluten-free diet. In Greece, a new neuropathic pain registry was created in 2014 in order to help gather data from real-world neuropathic pain patients. While still in its earliest phase, this registry has already produced demographic and treatment data that suggest suboptimal prescribing and less than recommended use of interventional procedures. Awareness campaigns are underway to encourage more Greek pain clinics to participate in this important registry.

## Introduction and background

Neuropathic pain was first defined as pain produced by aberrant activity generated within the nervous system itself by a neural lesion or other observable nervous system dysfunction. This original definition was subsequently modified to “pain caused by a lesion or disease of the somatosensory nervous system” [1]. This serviceable definition delineated the distinction between neuropathic and nociceptive pain, but since neuropathic pain could also be generated by physiologic maladaptive neuroplasticity, it was soon found inadequate [1]. This led to the recognition and definition of nociplastic pain [2] and proposals to subdivide specific categories of neuropathic pain [3]. Despite expert efforts at neuropathic nosology, the divisions among nociceptive, neuropathic, and nociplastic forms of pain remain somewhat blurred and sometimes overlap.

While current data derived from validated survey instruments estimated a 4% to 12% prevalence of neuropathic pain in the general population, it has been speculated that neuropathic pain and pain with a neuropathic component may be far more widespread [4]. Diagnoses are complicated, because pain patients may have nociceptive, neuropathic, and nociplastic pain at different pain sites or even co-existing in a single location of mixed pain [4].

This is a review paper based on presentations given at a specialty society meeting in Tunis, Tunisia on May 12-13, 2023, and reflects literature searches, the real-world clinical experience of the authors, and their insights.

## Review

Pathophysiology

How pain signals are generated and perceived differentiates nociceptive from neuropathic pain. In nociceptive pain, the noxious stimuli are transmitted by A-delta and C-fibers, whereby the initial sharp pain is transmitted by A-delta fibers, and the subsequent, duller and less-localized pain is transmitted by the C-fibers [5]. The ectopic discharges causing neuropathic pain originate at demyelination or regeneration sites on the nerve. These areas often exhibit an imbalance in the ratio of voltage-gated sodium channels (NaV) to potassium channels, such that with neuropathic pain there is a disproportionately higher number of NaV channels. Neuropathic pain can also exhibit alterations in how the α-adrenoreceptors function and how transient receptor potential (TRP) channels are expressed [6].

The nerve fibers that can be found in the peripheral nerves include the larger, myelinated, diameter A fibers (A-alpha and A-beta) and the smaller-diameter (C and A-delta fibers). C-fibers are unmyelinated whereas A-delta fibers are myelinated. Such small fibers may transmit pain or thermal perception signals whereas within the nerves there are also C-sympathetic fibers playing a role in the sweat gland innervation. The A-alpha fibers can be somatic motor or sensory transmitting signals about proprioception. The A-beta fibers are sensory transmitting signals regarding pressure, light touch, and vibration. Dysfunction of the A-alpha fibers transmitting signals of proprioception will lead to impaired balance (sensory ataxia). Dysfunction of the A-beta fibers will lead to reduced sensation regarding the relevant forms (i.e., pressure). Dysfunction of the thermal fibers (A-delta and C-fibers) may lead to impaired thermal sensation thresholds.

**Figure 1:**
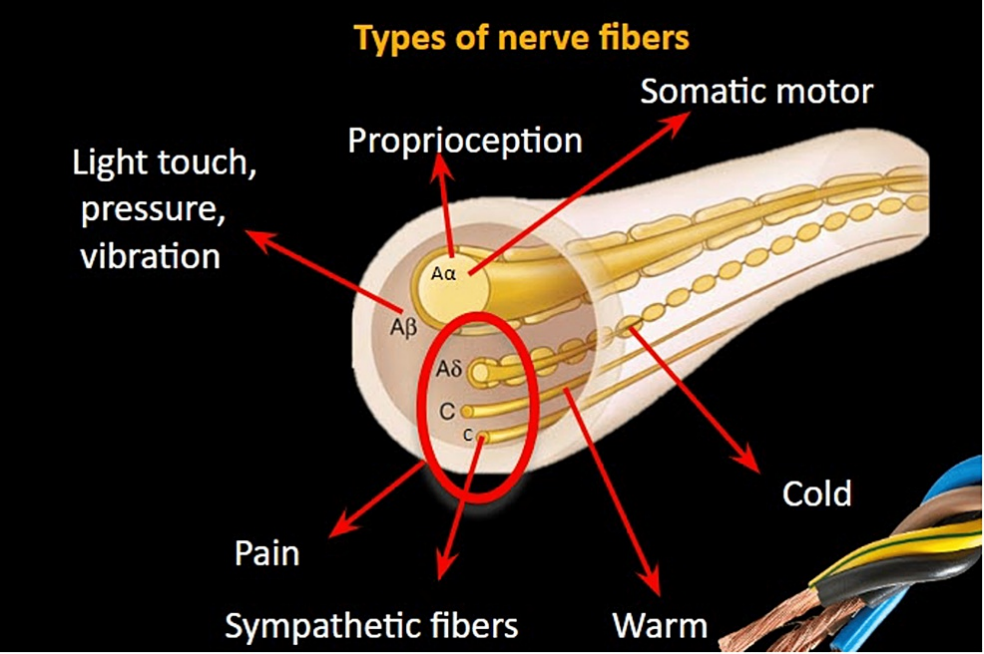
Types of nerve fibers and their main activities in a healthy individual. Original art courtesy of Professor Zis.

Peripheral neuropathic pain is often described as “burning” or “electrical” and may be accompanied by itchiness, tingling, and “pins and needles” that can occur as a result of small fiber involvement (A-delta and C-fibers). Figure 1 summarizes the main types of fibers that are present in a mixed peripheral nerve.

The paradoxical results of neuropathy are that it may cause a loss of sensation together with pain, even hypersensitivity, at the same time [7]. Nociceptive pain is the response to an exogenous stimulus, but neuropathic and nociplastic pain might better be considered as diseases. Damaged nerve fibers release local mediators, such as nerve growth factor (NGF) or tumor necrosis factor (TNF) which can cause damage to nearby and previously healthy nerve fibers [8]. The old term “central pain” refers to neuropathic pain caused by lesions of the central nervous system, such as post-stroke pain or pain with multiple sclerosis [8,9]. Peripheral forms of neuropathic pain are more common and include trigeminal neuralgia, postherpetic neuralgia, post-amputation pain, polyneuropathy, radiculopathy, and pain following peripheral nerve damage [9].

The neurons of the dorsal root ganglia (DRG) include several different types of NaV sodium channels, with distinct biophysical properties. There are several isoforms of NaV, which work together in the process of neuronal electrogenesis by generating action potentials for inter-neuronal communications [10]. Changes in DRG neuronal excitability are relevant to a variety of pathologies [11]. Multiple genes encode these numbered NaV channels for different functions, and their transcription and activation may be altered by injury or physiologic inputs [10]. NaV 1.1 through NaV 1.3 and NaV 1.6 through NaV1.9 are associated with electrogenesis and may be considered neuronal, while other NaV channels are not electrically excitable [12]. In a study of five patients with painful neuromas, it was found that NaV 1.3, NaV 1.7, and NaV 1.8 as well as an activated p38 subunit and ERK1/2 MAP kinases were expressed [13].

Dysregulation and mutations of the various NaV channels may result in hyperexcitable neurons, leading to acute or even chronic pain. Gain-of-function mutations in the NaV 1.7 channel can promote DRG hyperexcitability and lead to severe pain, while loss of the NaV 1.7 channels can blunt painful sensations [14]. There is also evidence that when healthy nerves are in contact with damaged or degenerated nerves, the healthy nerves may participate in pain signaling along with the damaged nerves [15].

The mysterious condition of phantom limb pain may be explained by the pathophysiology of neuropathic [16]. The central nervous system reorganizes itself with the loss of a limb with the goal of preserving maximum function. Thus, peripheral inputs from the limb, ectopic activity, neuroma, or other conditions may contribute to phantom limb pain. Psychological factors including stress and trauma may contribute as well, but their role and the role of sensory stimulation have not been elucidated. Cortical plasticity has been implicated in neuropathic pain in general and phantom limb pain in specific, because the body may preserve a cortical representation of the missing body part which may contribute to phantom limb pain [16]. Thus, the pathophysiology of neuropathic pain combines peripheral and central (spinal and supraspinal) mechanisms with changes induced by the inflammatory response.

Inflammation can promote and sustain neuropathic pain [17]. Immuno-active substances such as cytokines, neurotropic factors, and chemokines are released at the site of an injury, but if they initiate a large inflammatory cascade, inflammation may become a more generalized immune response. Neuroinflammation can activate the glial cells resident in the spinal cord and brain. Glial cells include astrocytes and oligodendrocytes (the macroglia), which are known to play a role in neuropathic pain. Microglia, the immune cells of the central nervous system, can release pro-inflammatory cytokines, such as interleukin and TNF, which propagate neuroinflammation further and are associated with painful symptoms. In fact, chronic neuropathic pain is closely associated with chronic neuroinflammation [17]. The many contributors to neuropathic pain include Schwann cells, satellite cells of the DRG, spinal microglia, and astrocytes to the extent that it has features similar to neuroimmune disorder [18]. Neuropathic and nociplastic pain are complex phenomena that are commonly seen in clinical practice. The core underlying mechanisms are shown in Table 1.

**Table 1:** The primary mechanisms underlying neuropathic and nociplastic painful symptoms. Table is original to Professor Tamburin.

Central mechanisms		Peripheral mechanisms	Inflammatory changes
Spinal	Supraspinal		
Spinal homosynaptic sensitization	Maladaptive cortical plasticity	Ectopic discharges in nociceptive fibers	Affecting nociceptive fibers
Spinal heterosynaptic sensitization	Psychological factors	Functional changes in the dorsal root ganglia	Affecting dorsal root ganglia
Changes in the descending inhibitory system	Contextual factors	Functional changes in intact nociceptive fibers	Affecting spinal cord
			Brain pain

Diagnosis and treatment of neuropathy

A diagnosis of neuropathic pain requires pain with a neuroanatomical distribution, a medical history that confirms or suspects a lesion or disease of the nervous system, and confirmatory testing [1]. There are no biomarkers for neuropathic pain and no gold standard for diagnosis [8]. Pain is largely self-reported by the patient. The clinical examination should explore diminished or absent tendon reflexes, sensory losses, and muscle weakness, or muscle atrophies. A variety of validated screening tools exist to interview patients about their symptoms.

The use of pain descriptors, such as “burning pain” or “electrical pain,” to characterize pain are used; however, verbal descriptions of painful sensations do not always map onto sensory tests [19]. Patients may struggle to describe pain in this way. Pain etiology is another diagnostic tool [19,20]. Since neuropathic pain is not usually relieved by nonsteroidal anti-inflammatory drugs (NSAIDs) or opioids, resistance to these drugs may also suggest that pain is neuropathic [7].

Symmetrical neuropathy is the most common presentation of peripheral neuropathy and is characterized by a reduced sensation in a length-dependent fashion that can lead to atrophy of distal muscles, particularly those of the feet [21]. On the other hand, a less frequently seen presentation is the asymmetrical sensorimotor neuropathy (mononeuritis multiplex), characterized by asymmetrical presentation, incremental deterioration, and pain with vasculitis being the underlying pathophysiological mechanism [22]. Asymmetrical sensory neuropathy (sensory neuronopathy), likewise uncommon, is characterized by patchy sensory loss and sensory ataxia [23].

To identify damaged nerves clinical assessment is valuable [24] but electrophysiological testing is very important. Patients presenting with chronic neuropathic symptoms will exhibit either pure small-fiber or large-fiber neuropathies, with or without small-fiber involvement. Large-fiber neuropathies can be either demyelinating or axonal [25].

Electrophysiological evaluation should begin with one upper limb and one lower limb. For the upper limb, the median, ulnar, or superficial radial nerve should be included, while for the lower limb, the tibial, peroneal, and sural nerve should be the objectives [26]. Axonal peripheral neuropathy may be diagnosed when nerve conduction studies reveal attenuated action potential amplitudes or when the sural to radial amplitude ratio (SRAR) is reduced.

With asymmetrical presentation both upper and lower limbs should be checked and electromyography may be helpful. The commonest asymmetrical neuropathy is sensory ganglionopathy, occurring as a paraneoplastic syndrome or in immune-mediated diseases like celiac disease. The proposed diagnostic criteria for sensory ganglionopathy include ataxia in upper or lower limbs, asymmetrical distribution patterns, sensory deficits not limited to the lower limbs, two or more nerves with abnormal nerve conduction in the lower limbs, and at least one sensory action potential or three sensory action potentials < 30% of the lower limit of normal in the upper limbs [27].

Causes of axonal neuropathy are numerous including infections, ischemia including vasculitis, metabolic disorders, exposure to toxins, genetic factors, and systemic diseases. Among the systemic diseases diabetes is the commonest risk factor but vitamin deficiencies, celiac disease, chronic renal disease, hypothyroidism, connective tissue diseases, paraneoplastic syndromes, critical illness polyneuropathy, paraproteinemias, and excessive consumption of alcohol are known risk factors.

Chronic idiopathic axonal polyneuropathy (CIAP) is a form of neuropathy usually with both sensory and motor length-dependent involvement in which there is axonal damage, slow disease progression commencing with an insidious onset, and exhibiting no clear etiology despite numerous investigations [28]. CIAP is not rare; it deserves greater attention due to the fact that it is both a frequent clinical presentation and a rare research subject. Men are more likely to have CIAP than women and the condition usually starts in the sixth decade of life with slow progression and plateaus [28]. Early-onset CIAP, defined as CIAP diagnosed before age 65, is rare [29].

A proposed diagnostic flowchart for axonal polyneuropathy appears below (Table 2).

**Table 2:** A proposed protocol for finding the cause of axonal polyneuropathy. Anti-ds-DNA, anti-double-stranded DNA; anti-ENA, anti-extractable nuclear antigen; c-Anca, cytoplasmic-antineutrophil cytoplasmic antibodies; HbA1C, hemoglobin A1c; HCV, hepatitis C virus; HIV, human immunodeficiency virus; p-ANCA, perinuclear anti-neutrophil cytoplasmic antibodies Table is original to Professor Zis.

Step	Action	Comments
PRIMARY CARE		
1	Patient history and presenting complaint(s)	
2	History of patient comorbidities, alcohol use, family history of neuropathy	Especially diabetes
3	Medication history and current use; history of exposure to toxins	
4	Clinical examination	
5	Basic laboratory tests	Full blood count, erythrocyte sedimentation rate, vitamin B12, folate. Fasting blood glucose, HbA1c, function tests for liver, kidney, thyroid. Celiac disease screening
SECONDARY/TERTIRARY CARE		
6	Advanced laboratory tests	Serum protein electrophoresis, serum angiotensin-converting enzyme, urinary Bence-Jones protein. Antinuclear antibodies, anti-neuronal antibodies, anti-ENA, anti-ds-DNA, rheumatoid factor, p-ANCA, c-ANCA, anti-HiV, anti-HCV
7	Imaging	Chest X-ray, skeletal survey, abdominal and chest CT, mammography, PET scan
8	Nerve biopsy	

Although all forms of neuropathy may cause pain, the proportion of patients who experience painful symptoms varies by type of neuropathy. Up to 70% of CIAP patients report neuropathic pain and neuropathy occurs in about half of all patients with paraneoplastic syndromes and at about the same rate in HIV [28,30-36].

Painful symptoms arising from various types of neuropathy have an adverse effect on quality of life [37]. While neuropathic pain is an important real-world clinical concern, only a subset of neuropathy patients respond to pharmacologic therapy [38-40]. Cannabinoids are currently being evaluated for their role in treating central neuropathic pain, but there have been a limited number of studies to date [38]. For all pharmacologic approaches to neuropathic pain management, it is important to consider that many of the recommended agents have dose restrictions, contraindications for many patients, and treatment-limiting side effects.

In a systematic review and meta-analysis, it was found from all studies reviewed that the number needed to treat (NNT) for neuropathic pain was 12.4 for antidepressants, 8.4 for gabapentinoids, 4.6 for selective sodium channel blockers, 6.9 for lidocaine, and 3.0 for cannabinoids. Note that the low NNT for cannabinoids might be at least partly attributable to the small number of studies [38].

Gluten neuropathy

Gluten neuropathy is a form of peripheral neuropathy that occurs in patients with serologically confirmed gluten sensitivity who may have enteropathy, that is, celiac disease. Gluten neuropathy is the second most common neurological manifestation of gluten sensitivity and/or celiac disease after cerebellar ataxia and encompasses multiple types of neuropathy, so an accurate neurological diagnosis is needed to ensure appropriate management.

Gluten sensitivity is serologically defined by the presence of antigliadin antibodies, anti-transglutaminase (TG2/TG6) antibodies, and/or anti-endomysial antibodies (EMA). In a study of 28,232 patients with celiac disease and 139,473 age- and sex-matched controls, it was determined that the presence of celiac disease could be associated with a significant risk for subsequent neuropathy; in fact, those with celiac disease were 2.5 times more likely to develop neuropathy than those without the celiac disease [41]. In most cases (~75%) gluten neuropathy is length-dependent neuropathy. In approximately 25% of cases, it is a sensory ganglionopathy [41]. In rare cases, gluten sensitivity may manifest exclusively as a neurological condition [42]. Note that gluten neuropathy patients may not have gastrointestinal symptoms [36]. Gluten neuropathy may be best defined as a neurological manifestation of gluten sensitivity, up to and including celiac disease [43].

Patients with celiac disease may have normal nerve conduction studies, but skin biopsies and thermal thresholds may be abnormal suggesting a pure small fiber involvement. In a case series of 13 patients with small fiber neuropathy due to celiac disease or gluten sensitivity, all patients reported pain. In fact, in more than half of the patients (62%), it was neuropathic pain that led to their diagnosis of celiac disease. Over the course of seven years, none of these patients progressed to suffer from large-fiber neuropathy [44].

An important complementary diagnostic test for patients with gluten neuropathy is the evaluation of the sudomotor function (sweat gland innervation). A cutaneous evaluation of electrochemical conductance is measured using a special device (i.e. Sudoscan™, Impeto Medical, Issy-les-Moulineaux, France) which can measure such sweat gland function in hands or feet [45]. Dysfunction of peripheral C sympathetic fibers affects about two-thirds of all patients with gluten neuropathy [43]. However, sudomotor dysfunction is not associated with a specific electrophysiological type of neuropathy, its severity, or whether or not the patient is on a strict gluten-free diet [43].

In a study of 60 gluten neuropathy patients, the prevalence of neuropathic pain was established to be 55%. Following a strict gluten-free diet was associated with significantly lowering the risk for neuropathic pain by almost 90% [36].

In a comparative quality of life study comparing 53 gluten neuropathy patients to age- and sex-matched controls, on the 36-Item Short Form Survey (SF-36) individuals with gluten neuropathy had worse scores than controls in functioning, role limitations due to physical health, and energy/fatigue. The gluten neuropathy group had significantly higher pain scores. People with gluten neuropathy on a gluten-free diet had higher quality of life scores than individuals with gluten neuropathy not on the diet [45].

Gluten neuropathy does not lead to severe disability. The majority of patients (about 80%) of those with gluten neuropathy could walk independently and only 5 to 10% may need bilateral support to walk. Very rarely if ever patients with gluten neuropathy are wheelchair-bound or bed-bound [46].

The importance of pain registries in neuropathic pain management

In today’s age of big data, local, national, and international registries have emerged as important foundational resources for clinical research, cost analyses, utilization data, and public health. The first patient registry was started in Norway in 1856 and it compiled data on patients with leprosy [47]. The foundational premise of the registry was to use observational methodology to gather standardized information (uniform data) on a particular population and follow that population over time [48]. Registries collect real-world data from very large sample sizes.

The utilization of registries is growing as their reputation for being valuable sources of information increases. While randomized clinical trials remain the “gold standard” in medical evidence, registries present a very interesting alternative. Like randomized clinical trials, they gather real-world information, but they do not have time limits imposed on their findings. A pain registry contains longer-term information than a 12-week pain trial. In specific, pain registries can identify and help quantify the prevalence of pain in specific populations and they provide data that may shed light on how effective specific analgesic regimens are [49]. Registries may provide valuable data that can help with professional education, healthcare administration and planning, early detection, disease prevention, epidemiologic data, therapeutic efficacy, drug development, and suggestions for research topics [49].

Interest in registries specific to pain is growing. The International Association for the Study of Pain (IASP) has convened a special interest group for pain registries; the National Institutes of Health in the United States is now running a national pain registry; and more specialized registries are being set up by organizations and societies [50-53]. Among these registries are the Swedish Quality Registry for Pain Rehabilitation, the Quebec Pain Registry, the international registry entitled PAIN-OUT, the Oslo University Hospital Pain Registry, and e-DOL, which started in 2019 in France and is supported by mobile devices [53].

There is an urgent and unmet medical need for a registry collecting information specifically on neuropathic pain. Among other things, a neuropathic pain registry would help determine what pharmacologic regimens were most effective for specific types of neuropathies. Neuropathic pain registries can be of particular value because the population of neuropathic pain patients is growing and there remain many unanswered questions about how to treat this condition. Neuropathic pain patients include the burgeoning number of cancer patients living with “managed disease,” postsurgical patients, people with painful diabetic peripheral neuropathy as well as other forms of neuropathic disorders or painful conditions with a neuropathic component [49]. Neuropathic pain has a profound impact on patients because it can increase depression and anxiety while adversely affecting sleep and function. Neuropathic pain has been associated with a 300% increase in healthcare resource utilization and it has staggering indirect costs because it increases absenteeism and decreases productivity at work [54]. In 2006, it was estimated that indirect costs due to neuropathic pain surpassed US$100 billion a year for the United States alone [55].

In 2014, the Hellenic Society of Pain Therapy and Palliative Care (PARH.SY.A) launched the first special registry for patients with chronic neuropathic pain. The goal was to register patients in Greece to determine how well their treatment adhered to guideline recommendations, the long-term safety and effectiveness of various drug therapies, and to improve care, patient management, and healthcare planning. A longer-term goal of this registry is to help guide and shape future clinical research [49].

Data were acquired in strict adherence to the principles of Registry Ethics in terms of informed consent, privacy, and data security. Using advanced analytics, the data could be explored in specific ways to identify the current status or explore specific topics. An important step in developing this neuropathic pain registry was to inform clinicians and other stakeholders about the registry and how it could be utilized to improve the clinical management of neuropathic pain. Current goals include using these data to develop studies evaluating the clinical efficacy of various treatments and other forms of retrospective research [56].

Registry users were all physicians and nurses specialized in pain care who applied to the Scientific Committee of the Registry. Data were collected only from patients who provided their informed consent, were suffering from a form of neuropathic pain, and were being treated for that pain in a pain or palliative care center in Greece. Using the PARH.SY.A website, a special portal was developed for the neuropathic pain registry. This pain registry site was to be streamlined, user-friendly, and easily accessible at any time from a computer, tablet, or smartphone. The clinician-user begins by entering baseline patient data or demographic information along with the patient’s medical history. The site allows delineation of the type of neuropathic pain: central, peripheral, cancer-related, or various neuropathic pain syndromes such as complex regional pain syndrome. Relevant lab work results and even a narrative text can be entered as well. The patient history incorporates the DN4 and Pain Detect questionnaire forms to help quantify neuropathic pain and pain locations, which can be drawn in on an interactive patient map. Treatments are recorded in terms of pharmacologic therapies, interventional approaches, and other treatments. A special tab was set up so the clinician can enter “current treatment” information distinct from previous discontinued therapies. The Greek pain registry was first submitted to the National Personal Data Protection Authority in 2014; it was approved and licensed in May 2016. The first entry occurred in June 2016, and by July 2020, there were entries from 24 different sites, providing data on 2,334 patients with neuropathic pain.

This early phase has already provided insights into the neuropathic pain patients in Greece. Most are female (65.4%), 57.9% are overweight or obese, 21.2% are current smokers and 13.3% are prior smokers. Most patients were self-referred to a neurologist (38%), while 27% were referred to a neurologist by a general practitioner, and 12% were referred by an oncologist. However, referrals came from a variety of sources, including diabetologists, dermatologists, orthopedic surgeons, and others. The clinical characteristics of these patients were that most had peripheral neuropathy (96.9%), with radiculopathy the single most common presentation (48.5%). Of the 3.1% with central neuropathy, the most common form was multiple sclerosis pain, followed by pain from stroke and spinal cord injury. It was noted that drugs sometimes changed after the patient was entered into the registry and more appropriate pharmacologic therapy was selected (Table 3). However, few patients were treated with interventional techniques such as nerve blocks, device-based therapies, or alternative treatments such as acupuncture. Psychological support was given to 8.1% of patients.

**Table 3:** Data from the Greek neuropathic pain registry for 2,332 neuropathic pain patients found that drug therapy after clinic visits showed a transition toward more appropriate medications. NSAIDs, non-steroidal anti-inflammatory drugs; COX-2, cyclooxygenase-2 These data are originally from the neuropathic pain registry [57], directly provided by Professor Rekatsina.

Drug	Percentage of patients who were administered this treatment BEFORE they came to the clinic	Percentage of patients who were administered this treatment AFTER they came to the clinic
Weak opioids	11.0%	55.3%
Pregabalin	7.8%	62.6%
NSAIDs	3.7%	4.1%
Duloxetine	3.4%	10.8%
Strong opioids	3.3%	22.3%
Gabapentin	2.1%	7.6%
Venlafaxine	1.2%	1.6%
COX-2 inhibitors	1.2%	17.7%
Lidocaine (patch)	0.9%	12.1%
Tricyclic antidepressants	0.6%	0.4%
Capsaicin (patch)	0.5%	1.0%

From the registry, it was found that pain scores over six visits decreased from 20.2 on the PainDetect scale at visit 1 to 13.5 on visit 6. The visual analog pain score was 7.1 on average at visit 1 and 5.0 at visit 6. Using the DN4 assessment, pain was 5.6 at visit 1 and 3.5 at visit 6. Using all assessments, pain generally decreased incrementally from visit 1 to visit 6.

Publications that emerged from the registry included one that found neuropathic pain patients often experienced a “diagnostic odyssey” or delay in receiving an appropriate diagnosis [58]. This diagnostic lag was three years from the onset of symptoms to the first consultation with the healthcare system and then nine more years within the system to find the accurate diagnosis and treatment. Another publication reported in an editorial that the registry was allowing for superior pain control and more efficacious planning for healthcare resources [49,57].

While the neuropathic pain registry allowed specialty centers in Greece to better follow national and international guidelines related to neuropathic pain care, the number of records entered into the system to date has been lower than expected. There are many plausible contributors to this lack of uptake: a small number of pain specialists, few specialized pain centers in hospitals, pain centers with limited days or hours of operation, and insufficient funding for this type of extra work for anesthesiologists and other clinicians. Of concern with the findings so far is the fact that interventional techniques are not used very often. From these findings, the goals for 2023 have emerged: recruiting more pain centers, increasing referrals, adding more outcome measures related to sleep, quality of life, etc., and gaining acceptance from the competent national authorities. A more clinical goal will be to determine why interventional procedures are not used as frequently as one might expect, despite their potential efficacy and safety.

Discussion

Neuropathic pain represents a major clinical challenge to patients, clinicians, and the healthcare system. The years-long “diagnostic odyssey” endured by some patients and the lack of a “gold standard” of care for neuropathic pain has resulted in under-treated neuropathic pain symptoms, reduced quality of life, and decreased function [58]. Most neuropathic pain is treated with combination therapy but the optimal combination is not known and the agents used for this care often have treatment-limited side effects. The role of interventional pain care in treating neuropathic pain is an area of great interest and potential. Nerve blocks and device-based pain therapy may be helpful in treating certain types of neuropathic pain, but there are only a few studies to help guide clinical decision-making [59]. New drug targets may result in new agents for neuropathic pain control, such as the sigma receptors [60].

A challenge in addressing neuropathic pain is its many forms. Treatment of trigeminal neuropathic pain, for example, differs from treatment for painful peripheral diabetic neuropathy [61]. Inhibition of the voltage-gated sodium channels may play an important role in new pharmacologic regimens or at least a better understanding of neuropathic pain mechanisms.

## Conclusions

There is a large and burgeoning population of neuropathic pain patients, many of whom may live years or the balance of a lifetime with neuropathic pain symptoms. With the growing population of patients living with managed diseases such as cancer or multiple sclerosis and advances in treating spinal cord injury, there is a large and growing unmet medical need for better diagnosis and treatment of neuropathic pain. The development of neuropathic pain registries, such as the one in Greece, can provide valuable information about how neuropathic pain presents and how various patient populations respond to treatment.
